# Can We Grow Valves Inside the Heart? Perspective on Material-based In Situ Heart Valve Tissue Engineering

**DOI:** 10.3389/fcvm.2018.00054

**Published:** 2018-05-29

**Authors:** Carlijn V. C. Bouten, Anthal I. P. M. Smits, Frank P. T. Baaijens

**Affiliations:** ^1^Soft Tissue Engineering and Mechanobiology, Department of Biomedical Engineering, Eindhoven University of Technology, Eindhoven, Netherlands; ^2^Institute for Complex Molecular Systems (ICMS), Eindhoven University of Technology, Eindhoven, Netherlands

**Keywords:** endogenous regeneration, biomaterials, host response, tissue remodeling, clinical translation

## Abstract

In situ heart valve tissue engineering using cell-free synthetic, biodegradable scaffolds is under development as a clinically attractive approach to create living valves right inside the heart of a patient. In this approach, a valve-shaped porous scaffold “implant” is rapidly populated by endogenous cells that initiate neo-tissue formation in pace with scaffold degradation. While this may constitute a cost-effective procedure, compatible with regulatory and clinical standards worldwide, the new technology heavily relies on the development of advanced biomaterials, the processing thereof into (minimally invasive deliverable) scaffolds, and the interaction of such materials with endogenous cells and neo-tissue under hemodynamic conditions. Despite the first positive preclinical results and the initiation of a small-scale clinical trial by commercial parties, in situ tissue formation is not well understood. In addition, it remains to be determined whether the resulting neo-tissue can grow with the body and preserves functional homeostasis throughout life. More important yet, it is still unknown if and how in situ tissue formation can be controlled under conditions of genetic or acquired disease. Here, we discuss the recent advances of material-based in situ heart valve tissue engineering and highlight the most critical issues that remain before clinical application can be expected. We argue that a combination of basic science – unveiling the mechanisms of the human body to respond to the implanted biomaterial under (patho)physiological conditions – and technological advancements – relating to the development of next generation materials and the prediction of in situ tissue growth and adaptation – is essential to take the next step towards a realistic and rewarding translation of in situ heart valve tissue engineering.

## Introduction

Since the introduction of the first artificial aortic heart valve by Hufnagel et al. more than six decades ago (1), heart valve prosthesis design has seen revolutionary changes in the endeavor to reduce prosthesis-related complications and to treat diverse patient groups. These include the development of bio-prostheses consisting of preserved human or animal tissue (2, 3) and the recent introduction of valve designs for transcatheter valve replacement (4). A true paradigm change, however, has been the construction of living valves through the process of tissue engineering. Conventional tissue engineering, also named *in vitro* tissue engineering, is defined as the culture of cells – preferably from an autologous source – in combination with a degradable scaffold, to create a living implant or a living tissue mimic outside the human body (5). Living heart valve prostheses offer the potential to grow and adapt to changes in physiological demand and, as such, can last a lifetime. This was conceived as the holy grail for pediatric patients and the increasing number of patients with “grown up congenital heart disease” (GUCH), who will need one or more heart valve replacements later in life (6). Despite encouraging exemplary results (7, 8) and numerous modifications to the procedure (9–12), however, clinical translation has proven difficult. This is primarily caused by suboptimal long term *in vivo* results due to cell traction, consequent valve leaflet retraction, and unforeseen host responses to the constructs after implantation (13–16). In addition, clinical translation is hindered by the logistic and regulatory complexity of the procedures, very limited shelf life, and costly cell and tissue culture in specialized laboratories, restricting the therapy to developed Western countries (17). These drawbacks have led clinicians and scientists to wonder if heart valve tissue engineering (HVTE) will ever make a difference in heart valve replacement therapy (18).

### In Situ Heart Valve Tissue Engineering

Inspired by the *in vivo* host response of living tissue engineered valves, and to resolve the issue of cell traction-induced leaflet retraction, the concept of *in situ* HVTE using acelluar starter matrices is explored by different groups (See Table 1). For instance, de-cellularized *in vitro* engineered heart valves have been developed (56, 57). This approach aims at the creation of a living valve at the site of implantation using a cell-free, yet *in vitro* cultured, extracellular matrix that recruits endogenous cells after implantation. In contrast to de-cellularized xenografts and homografts (19, 27, 28, 58) *de novo* engineered matrix valves do not depend on the availability of a donor valve or tissue. These *de novo* engineered matrices show rapid repopulation with host cells required for growth and remodeling, both in sheep and non-human primates (45, 46, 48, 49). As such, the outlooks for clinical application are promising, but creation of these valves is still laborious and costly.

**table 1:** Selection of (pre)clinical studies on *in situ* tissue engineered heart valves.

Material type	Model	Main findings/status	Refs.
**Decellularized allografts**			
Decell. allografts	PV and AV replacements in ovine and porcine models	Less calcification and improved durability compared to cryopreserved valves. Adequate nctionality demonstrated in juvenile, growing sheep, as well as elderly sheep. Cellularization typically persistent but partial.	(19–25)
Decell. allografts	PV replacement in children and young adults	Improved freedom from reoperations. Partial cellularization of the leaflet. No systemic immune response.	(26–28)
Decell. allografts + collagen conditioning treatment	PV replacement in baboons and growing lambs	Decreased antigenicity and improved somatic growth potential by collagen conditioning treatment.	(29, 30)
**Decellularized xenografts**			
Decell. xenografts (porcine)	PV replacement in adults and children	Mixed clinical results. Recellularization potential and immunological compatibility seems strongly dependent on decellularization and cryopreservation methods.	(31–34)
Decell. xenografts + various functionalizations	PV replacement in ovine and canine models	Various functionalization treatments to improve *in situ* recellularization, including CD133ab, HEP/HGF, G-CSF.	(35–37)
Decell. xenografts + PHB coatings	PV and AV replacements in sheep	Hybrid polymer-coated decellularized xenografts to improve mechanical and structural properties.	(38, 39)
**Decellularized ECM**			
Decell. SIS (CorMatrix)	Various valve replacements (PV, AV, MV) in children and adults	Mixed immunological response of remodeling and inflammation. Reports of severe insufficiency and degeneration. Consistent reporting of no remodeling into the typical 3-layered valve structure.	(40–42)
Decell. SIS (CorMatrix)	TV replacement in pig	*In situ* cellularization and remodeling reported, with potential for growth. Severe paravalvular regurgitation.	(43, 44)
**Decellularized *de novo* tissue-engineered heart valves**			
Decell. homologous TEHVs	Minimally-invasively implanted PV in sheep and non-human primates	Decellularized TEHV technology compatible with minimally-invasive valve delivery. Extensive *in situ* cellularization of leaflets and tissue remodeling, including elastogenesis. Leaflet retraction and regurgitation at >8 weeks follow-up.	(45–47)
Decell. tubular TEHVs	Implantation as AV in sheep and PV in growing lambs	Extensive cellularization of leaflets and tissue remodeling, including elastogenesis. Sustained functionality for 6-months as AV. Progressive regurgitation of PVs in growing lambs.	(48, 49)
**Resorbable synthetic valves**			
PGA/P4HB, on-the-fly preseeded with BMCs	Transapically delivered AV in sheep and PV in non-human primates	Feasiblity of technology demonstrated with acute valve functionality. Rapid polymer resorption	(50, 51)
P4HB/gelatin hybrid	Transapically delivered PV in sheep	Feasiblity of technology demonstrated with acute valve functionality.	(52)
Slow-degrading supramolecular elastomers	PV and AV replacements in sheep	Sustained 1-year functionality with extensive *in situ* cellularization and tissue formation. Proof-of-concept for *in situ* TEHV using resorbable synthetic valves. Compatible with minimally-invasive delivery in PV and AV positions.	(53–55)
Slow-degrading supramolecular elastomers	PV replacements in pediatric patients	First ongoing clinical trials using resorbable synthetic valves (Xeltis XPlore-I and XPlore-II, NCT numbers: NCT02700100, NCT03022708).	-

AV, aortic valve; BMCs, bone marrow-derived cells.; G-CSF, granuloctye colony stimulating factor; HEP, heparin; HGF, hepatocyte growth factor; MV, mitral valve; P4HB, poly-4-hydroxybutyrate; PGA, polyglycolic acid; PHB, polyhydroxybutyrate; PV, pulmonary valve; SIS, small intestine submucosa; TEHV, tissue-engineered heart valve; TV, tricuspid valve.

**Figure 1:**
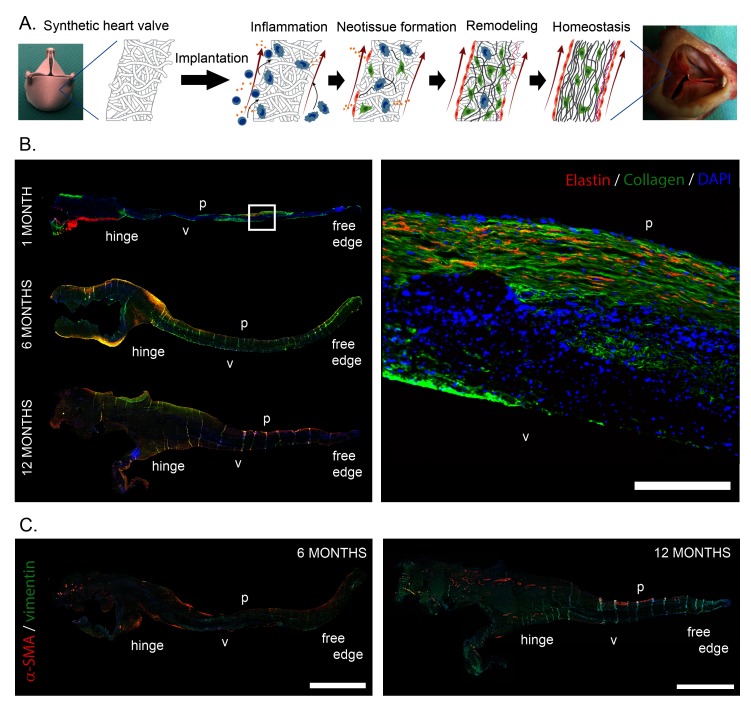
**(A)** Schematic representation of the hypothesized sequence of events, transforming an acellular synthetic valvular scaffold into an autologous, living heart valve *in situ*. After implantation, the implantation of the foreign material triggers an inflammatory response, which subsequently induces the formation of new tissue, while the initial synthetic scaffold is being degraded. Ideally, the newly formed tissue is remodeled to attain the well-organized native-like three-layered structure of the valve, with the goal of achieving tissue homeostasis. B. Neo-matrix formation in the leaflet of an *in situ* engineered heart valve, 1 month, 6 months and 12 months post implantation, grown from a slow degrading electrospun elastomeric (bis-urea-modified polycarbonate) scaffold in the pulmonary position in sheep. A layered structure containing cells (DAPI, blue), and collagen (green) co-localized with elastin (red) can be observed in the longitudinal sections. C. Cellularization of *in situ* engineered valves with predominantly vimentin (green) expressing mesenchymal cells and decreasing presence of α-smooth muscle actin (red) expressing active myofibroblast-like cells from 6 to 12 months after implantation. p: pulmonary side; v: ventricular side of the leaflet. Scale bars represent 200 µm **(B)** and 1 mm **(C)**. Subfigures A and C are adapted and reprinted from (55) with permission from Elsevier. Subfigure B is courtesy of Sylvia Dekker, Eindhoven University of Technology.

In recent years the use of biodegradable synthetic starter matrices has emerged as an alternative technology to grow living valves inside the heart (59). This technology offers readily available valvular grafts at substantially reduced costs. Porous synthetic polymer scaffolds are attractive candidates for the procedure as they can be rationally designed to accommodate cell recruitment and orchestrate tissue formation, while maintaining valve functionality. The technology is compatible with current regulatory frameworks for medical devices and artificial heart valves and exquisitely suited for both surgical and transcatheter valve delivery. We have investigated *in situ* HVTE using a slow-degrading electrospun bis-urea-modified polycarbonate elastomeric graft (55). When implanted as a surgical pulmonary valve replacement in sheep, valves maintained hemodynamic performance over a 12-month follow-up period as endogenous cells that produced a native-like, layered extracellular matrix slowly replaced the graft (Figure 1B). Transapically delivered pulmonary valves in Nitinol stents showed similar native-like matrix formation and good hemodynamic performance over a 6-month follow-up period.

Although this concept is widely explored for *in situ* engineered vascular grafts, leading to exciting preclinical and clinical trials (e.g., reviewed by 60 and 61), this was the first long-term pre-clinical proof of concept that *in situ* formation of living valvular tissue is possible without the use of any donor tissue or even *in vitro* cell and tissue culture. In parallel to – and independent of – this scientific proof of concept ongoing commercial developments of biodegradable polymer pulmonary valves have recently led to a small-scale clinical trial in pediatric patients (Xplore-I and Xplore-II trials) as well as the preclinical exploration of transcatheter aortic heart valves (62).

Despite these developments, complete understanding of neo-tissue formation is missing. In addition, growth of *in situ* engineered heart valves has not been demonstrated yet. Next to ongoing long-term *in vivo* investigation of the technology, a number of scientific and technological challenges must be addressed before *in situ* HVTE can be translated into a routine clinical practice. Below, we highlight the most critical issues.

## OUTSTANDING CHALLENGES

### I Understanding Materials-Driven Regeneration

Regenerative medicine in general – and *in situ* tissue engineering in particular – builds on the intrinsic self-healing and regenerative capacity of the human body. Hence, for *in situ* HVTE to be successful and safe, our understanding of the intelligent and diverse ways of human tissue adaptation and regeneration in response to a non-living degrading biomaterial under hemodynamic conditions is critical. Since this knowledge is virtually missing, the prime challenge is to develop a mechanistic understanding of materials-driven valve regeneration and unveil the potential and limitations of *in situ* HVTE under various (patho)physiological circumstances.

The core concept of *in situ* HVTE is that a degradable synthetic heart valve scaffold transforms into viable tissue with growth potential via an inflammatory response to the scaffold (Figure 1A). While little experimental data regarding the fundamental inflammatory and regenerative processes underlying *in situ* HVTE is available, mechanistic data from developmental biology and other *in situ* TE applications may give us more insight into these processes, as reviewed in more detail elsewhere (63). Specifically, studies employing resorbable vascular grafts have demonstrated that the host response to such an implanted grafts in the bloodstream is a cascade of events, initiated by the acute inflammatory response (64).

Upon implantation, the scaffold is first and foremost colonized by immune cells from the bloodstream (e.g., granulocytes, monocytes), followed by recruitment of progenitor cells, macrophages, lymphocytes, and tissue forming cells from blood and adjacent tissue, which are attracted by inflammatory cytokines and chemokines expressed by the immune cells. Next, the scaffold is degraded by foreign body giant cells while endogenous extracellular matrix is produced. Studies on highly regenerative species, such as axolotls, zebrafish and African spiny mice have demonstrated that macrophages are critical for regeneration (65–67). Similarly Hibino et al. demonstrated that systemic macrophage depletion led to a complete abrogation of regeneration of *in situ* TE vascular grafts in mice (68).

By coordinating the initial infiltration and differentiation of innate immune cells into the scaffold, the inflammatory response can potentially be harnessed to avoid chronic inflammation and tissue fibrosis (69). While the role of macrophage polarization in heart valve regeneration remains to be elucidated, it has been postulated that the differentiation of monocytes towards a regenerative macrophage (M2) phenotype should be enhanced early in the process to create the prerequisite initial conditions for stable tissue formation (70, 71). Additionally, recent data on the biomaterial-driven regeneration of skeletal muscle revealed an essential role for T helper 2 cells in the macrophage-driven regeneration (72). Following these initial processes, graft endothelialization and functional matrix organization (i.e., anisotropy, layered-ness) must be achieved, while preventing adverse effects like neo-intima hyperplasia, valvular fibrosis and calcification. The exact origin of the colonizing mature tissue cells remains speculative. With respect to endothelialization, studies in rodents have suggested transanastomotic ingrowth as the primary source of endothelial cells (73). However, the relevance of this suggestion for the human scenario has been contested, and recently transmural capillary ingrowth has been indicated as the primary route of endothelialization (74).

Our own preclinical results have indeed verified the above processes when using macro-porous, degradable electrospun scaffolds. Upon implantation the scaffolds were immediately colonized by immune cells from the bloodstream, followed by recruitment of macrophages and tissue forming myofibroblast-like α-SMA^+^ and fibroblast-like vimentin^+^ cells from blood and adjacent tissue (valvular root) to eventually achieve a stable, quiescent α-SMA^−^/vimentin^+^ valvular interstitial-like cell phenotype (Figure 1C). In addition, a layered ECM was developed, with mature collagen and elastin fibers, covered by a confluent endothelium weeks to months after implantation (21, 75). It remains to be elucidated if *in situ* tissue development will be similar under more demanding conditions, such as in case of aortic valve replacement.

Systematic analysis of immune cell recruitment and polarization in preclinical studies, relevant for profound mechanistic understanding, requires the development of species-specific markers (76). More importantly, innate and adaptive immune responses may differ among species (77–79), strongly reducing the impact and translation of preclinical observations for human insights. For translational purposes it should furthermore be noted that the inflammatory host response and subsequent matrix formation is different in young versus old patients (80, 81), and can be affected by common comorbidities, like diabetes or kidney disease, common in older patients requiring heart valve replacements (82–85). Finally, it is far from clear if and how *in situ* tissue regeneration can be controlled under conditions of genetic or acquired disease.

In order to deal with the above-mentioned inter-species and inter-patient variability in the processes of material-driven inflammation and regeneration, the development of dedicated models is paramount. *In vitro* engineered laboratory models, based on human cells (either healthy or diseased) can be exploited to gain an initial understanding of tissue integration and remodeling in response to scaffolds (e.g., reviewed by 86 and 87). Dynamic *in vitro* co-culture platforms are eminently suitable to screen the interactions between human (circulatory) immune cells and valvular scaffolds under physiologically relevant hemodynamic stimuli, such as shear stress (88–90) and cyclic strains (91, 92). By using primary patient-derived cells, the influence of patient-specific characteristics on the cell-scaffold interactions can be assessed (e.g., 93, 94) Accordingly, preclinical animal models are increasingly being tailored to match specific clinical scenarios, for example by considering age (35), induced pathologies (38), or by using humanized animal models (95) or genetically modified animal models e.g., via CRISPR technologies (96, 97). All in all, the development of such refined, more personalized *in vitro* and *in vivo* models enables the fundamental unraveling of materials-driven regeneration for a wide range of patient populations.

### II Biomaterial Development and Rational Scaffold Design

Although the use of synthetic degradable materials as valve replacement is attractive from a clinical perspective, the success of this approach fully depends on the generation of sophisticated biomaterials and the processing thereof into valvular scaffolds. For secured valve functionality, these scaffolds should: (i) take over valve functionality immediately upon implantation, thus providing structural and mechanical support; (ii) fully interact and integrate with their biological environment, instructing and guiding neo-tissue formation by providing a microenvironment with the necessary biochemical and biophysical cues for cells to home, stabilize, synthesize, and organize their own load-bearing extracellular matrix. (iii) maintain tissue functionality at all times, thus degrading in pace with neo-tissue formation and permitting matrix homeostasis and remodeling to evolving functional demands; and (iv) result in completely endogenous and well-structured, layered and endothelialized valves that can adapt to somatic growth.

These demands are relevant across lengths scales. For instance, valve functionality (opening and closing, load-bearing properties) is determined by macroscopic mm-cm scale properties of the valvular scaffold, such as valve geometry, while cell behavior is mainly dependent on microscale properties, like porosity or chemical composition of the scaffold. Degradation profiles, on the other hand, will affect both microscopic and macroscopic properties.

Nowadays, many biomaterials and scaffolds are designed to induce tissue formation or even regeneration through direct interactions with proteins and cells via e.g., chemical function and binding affinity, but also via biophysical properties, like stiffness and nano-, micro- and mesoscale topologies (98–100). Revolutionary improvements in materials science, especially in the area of supramolecular polymers (101, 102) have recently resulted in the development of a new class of biomaterials that can be rendered bioactive and bioresponsive via the appending of functional moieties and tuned with respect to mechanical properties and degradation rate/mechanisms through simple “mix-and-match” assembly. These dynamic materials can interact with the biological system in an almost natural way; instructing and responding to cells and offering full control over the cellular environment. At the same time, they can be used to restore large defects, while providing temporary mechanical and structural support. Recent results with SDF-1α functionalized scaffolds, for instance, demonstrate the potential of these materials in the cardiovascular system (103).

A main challenge is to develop instructive materials that are capable of harnessing the inevitable host response, for instance by selective recruitment of immune cells or by skewing macrophage polarization. Previous studies indicate that macrophage polarization in cell-free scaffolds can be achieved via the release of specific cytokines and trophic factors (MCP-1, SDF1α, bFGF; 64, 68, 104). More recent findings, however, demonstrate that the biophysical microevironment (strain, shear stress, anisotropy) experienced by infiltrating monocytes suffices to modulate macrophage polarization (44, 51, 105). As this would prevent the use of bioactive moieties, the processing of materials into scaffolds with the right initial microstructure might suffice to control the delicate balance between fibrotic and regenerative tissue formation.

Valvular scaffolds have been processed from a wide range of synthetic biomaterials (106, 107) using processing methods like electrospinning (108, 109), 3D printing (110), direct write melt electrospinning (111), jet spinning (52), and double component electrodeposition (112) to control valve macro and microstructure. The outcomes of these studies suggest that controlling leaflet shape and thickness, as well as pore size (for rapid cell repopulation, 113) are among the most critical parameters for ultimate valve function and regeneration.

Still, scaffold development for *in situ* HVTE would benefit greatly from systematic studies on the effects of individual and combined micro and microscale properties on valve function and regeneration. These should include currently unexplored properties like blood-scaffold interactions under anticoagulation therapy (114) and antimicrobial properties (115). The systematic studies may take advantage from the above-mentioned *in vitro* models for screening candidate materials and even move towards the development of personalized scaffolds. Given the myriad of possible combinations, however, high-throughput analysis techniques combined with data mining may be a faster option (116, 117).

### III Predicting Tissue Development and Growth

Computational modeling can also accelerate scaffold design across length scales. A significant example is the development of a predictive computational model to generate new testable hypotheses for scaffold properties that favor tissue engineered neovessel formation and function (118). For HVTE such models are scarce but indispensible. Initially, computational analysis focused at the biomechanics of heart valves and was directed at understanding the stress and strain distribution in the valve leaflets and valve root in relation to the geometry and mechanical properties of the tissues (e.g., reviewed in 119, 120). In particular, the impact of the collagen fiber architecture on the deformation patterns was investigated (121, 122). To this end, constitutive models with increasing complexity where developed to capture the microstructure of the valve. Upon the development of dedicated fluid-structure interaction algorithms, these models could also be used to investigate the impact of the microstructure on the opening and closing behavior of the valve leaflets (123). It was found that collagen fiber architecture not only significantly impact tissue stresses and strains during diastole, their predominant circumferential orientation also has a large effect on valve opening during systole and contribute to the stability of valve motion (124). These observations are likely relevant for valvular scaffolds as well and can be translated into “scaffold leaflets” with a predominantly circumferential anisotropy.

Understanding remodeling of the fibrous collagen network in response to static and dynamic loads – relevant for (neo)tissue adaptation and homeostasis – has evolved significantly over the years. To provide for a mechanistic understanding, these models include collagen synthesis and degradation profiles, as well as the impact of cellular traction forces resulting from intracellular actin stress fibers (125). Recently, these models have been calibrated against a number of experimental observations, demonstrating a remarkably accurate description of the collagen remodeling in native heart valves (126). Yet, they also reveal the complexity of the interplay between valve geometry, the evolving structural and mechanical properties of the tissue, and traction forces generated by the cells, thereby demonstrating the grand challenges in predicting neo-tissue formation and homeostasis in scaffold-driven *in situ* HVTE.

When using a fibrous scaffold as a starter matrix for *in situ* tissue engineering, computational models can provide the* initial* guidelines with respect to the geometry, mechanical properties, and – in particular – the fiber alignment that controls the degree of anisotropy of the leaflets (127). It is the combination of these properties that determines the deformation patterns in the leaflets that, together with the contact guidance provided by the fibers, dictates the alignment of the endogenously synthesized collagen network (128), and thereby the mechanical functionality of the valve (129).

The next modeling challenge will be the analysis of evolving neo-tissue formation under various scaffold degradation profiles. Our preclinical studies have shown several stages in the process of tissue formation (55). Next to the deposition of collagen and elastin fibers inside the scaffold, significant tissue formation on top of the scaffold is observed, and with time a layered architecture develops. In regions with (near) complete scaffold degradation the tissue composition is markedly different from those with incomplete scaffold degradation. To analyze this staged tissue formation, advanced analysis tools are needed that not only account for mechanical cues, but also for cell signaling mechanisms driven by these cues to describe the complicated processes of growth and remodeling and to predict tissue self organization *in situ*. For example, it has been shown that Notch signaling has a profound impact on the layered architecture in heart valves and new models should incorporate this signaling (130). When established, such models may be extended with more (and even genetically affected) signaling pathways to provide insights in the requirements for scaffolds that drive tissue formation and ultimately tissue stability and functionality in a variety of pathological conditions.

## Clinical Perspective

Today, the question remains whether HVTE will ever make a difference. Yet, significant progress has been made and different concepts are being prepared for translation to the clinic (131). We have no doubt that material-based *in situ* HVTE will leave its footprint on the ongoing quest for a living heart valve replacement. Albeit scientifically and technically extremely challenging, the *in situ* approach may be more attractive to apply in clinic than other tissue engineering approaches as it will eliminate cell and tissue culture, can be easily scaled up to therapeutic needs, and may be developed into personalized therapies, while at the same reducing regulatory complexity. As such, the approach can bring living valve replacement therapy to many patients worldwide and will not just cater to the wealthy.

Obviously, tackling the above challenges will determine whether we reach this goal, or whether *in situ* HVTE will remain an academic exercise. A combination of multidisciplinary research – unveiling the mechanisms, potential and limitations of the postnatal human body to adequately respond to the implanted biomaterial scaffold – and technological advancements – relating to scaffold development and the prediction of tissue adaptation under various conditions – is essential to take the next step en route to clinical application. This step should include rigorous and extensive preclinical evaluation in direct comparison with *in vitro* and *in silico* studies to scrutinize and optimize the technology. Next, a number of reliable, well-regulated randomized clinical trials should be performed for which standardized procedures and endpoints are defined (132). In parallel, simulation models should be developed that estimate the quality of life of patients as well as cost-effectiveness of the new technology compared with existing valvular replacement therapies. These measures will support decision makers in their authorization strategy and will aid patients and doctors in their choice of a prosthetic valve (133), thereby contributing to a cautious, realistic, and rewarding clinical translation.

## Author Contributions

CB suggested the subject of the review and drafted the outline of the manuscript. CB, AS, and FB drafted and edited the contents of the manuscript.

## Conflict of Interest Statement

CB and FB are non-voting shareholders of XELTIS AG based on intellectual property licensed to XELTIS. The authors declare that the research and the work related to the preparation of this manuscript was performed in the absence of any commercial or financial relationships.
